# Coping Strategies Influence Cardiometabolic Risk Factors in Chronic Psychological Stress: A Post Hoc Analysis of A Randomized Pilot Study

**DOI:** 10.3390/nu14010077

**Published:** 2021-12-24

**Authors:** Deborah Armborst, Norman Bitterlich, Birgit Alteheld, Daniela Rösler, Christine Metzner, Roswitha Siener

**Affiliations:** 1 Department of Urology, Medical Nutrition Science, University Hospital Bonn, Venusberg-Campus 1, 53127 Bonn, Germany; Roswitha.Siener@ukbonn.de; 2 Department of Biostatistics, Medicine and Service Ltd., Boettcherstr. 10, 09117 Chemnitz, Germany; bitterlich@medizinservice-sachsen.de; 3 Department of Nutrition and Food Sciences, Nutritional Physiology, University of Bonn, Nussallee 9, 53115 Bonn, Germany; b.alteheld@uni-bonn.de; 4 Bonn Education Association for Dietetics r. A., Fuerst-Pueckler-Str. 44, 50935 Cologne, Germany; office@bfdev.de (D.R.); christine.metzner@rwth-aachen.de (C.M.); 5 Clinic for Gastroenterology, Metabolic Disorders and Internal Intensive Medicine (Medical Clinic III), RWTH Aachen, Pauwelsstr. 44, 52074 Aachen, Germany

**Keywords:** stress management, perceived stress questionnaire (PSQ_30_), psychological neurological questionnaire (PNF), visceral adiposity, fatty liver index (FLI), cardiometabolic risk, hypothalamic–pituitary–adrenal (HPA) axis, coping strategies, stress habituation

## Abstract

Chronic psychological stress can result in physiological and mental health risks via the activation of the hypothalamic–pituitary–adrenal (HPA) axis, sympathoadrenal activity and emotion-focused coping strategies. The impact of different stress loads on cardiometabolic risk is poorly understood. This post hoc analysis of a randomized pilot study was conducted on 61 participants (18–65 years of age) with perceived chronic stress. The Perceived Stress Questionnaire (PSQ_30_), Psychological Neurological Questionnaire (PNF), anthropometric, clinical and blood parameters were assessed. Subjects were assigned to ‘high stress’ (HS; PSQ_30_ score: 0.573 ± 0.057) and ‘very high stress’ (VHS; PSQ_30_ score: 0.771 ± 0.069) groups based on the PSQ_30_. Morning salivary cortisol and CRP were elevated in both groups. Visceral adiposity, elevated blood pressure and metabolic syndrome were significantly more frequent in the HS group vs. the VHS group. The fatty liver index (FLI) was higher (*p* = 0.045), while the PNF score was lower (*p* < 0.001) in the HS group. The HS group was comprised of more smokers (*p* = 0.016). Energy intake and physical activity levels were similar in both groups. Thus, high chronic stress was related to visceral adiposity, FLI, elevated blood pressure and metabolic syndrome in the HS group, while very high chronic stress was associated with psychological–neurological symptoms and a lower cardiometabolic risk in the VHS group, probably due to different coping strategies.

## 1. Introduction

Chronic psychological stress is increasingly recognized as a significant contributor to mental and physiological disorders in modern societies [1,2,3]. The term ‘stress’ is used to describe physiological and behavioral responses to real or perceived intrinsic or extrinsic stimuli or ‘stressors’ threatening homeostasis [4,5]. The brain is the central organ of the perception and the response to stress. It operates as part of a complex and non-linear network via the sympathetic and parasympathetic systems, the hypothalamic–pituitary–adrenal (HPA) axis, the immune system, metabolic hormones and molecular processes within all organs to promote adaptation (‘allostasis’) to protect the body. However, exposure to perceived chronic stress leads to an overuse of these mediators, and adaptation to the chronicity can result in persistent dysregulation that contributes to stress-related pathophysiology (‘allostatic load’) [6,7,8,9,10].

The individual response to chronic stressors and resulting disorders depends on numerous factors such as genetic predisposition, stress intensity, stress susceptibility, neural processing, and subsequent compensatory adjustments, social environment, coping strategies, as well as the chronicity of exposure [10,11,12,13]. Yet, there is little agreement about what aspects and dimensions of the complex construct ‘stress’ matter most for human health and disease [14]. Major life stressors involving interpersonal stress, social rejection, combinations of high mental demands and low decision latitude as well as high efforts and low rewards are considered to be among the strongest prospective psychosocial risk factors for mental disorders [15,16]. In Germany, sleep disturbances, diagnosed burnout syndrome, and depressive symptoms were much more frequent in people with a high chronic stress level than in those without high levels of stress [17]. Likewise, in a working age population in Sweden [18] and in female undergraduates in the United States of America [19], a higher level of perceived chronic stress was often accompanied by symptoms of depression and/or anxiety. In comparison with these results, Sara et al. [20] showed that individuals with chronic job strain, effort–reward imbalance, or organizational injustice are at an increased risk of cardiovascular diseases (CVD), directly or through mediating factors such as hypertension, high cholesterol, or maladaptive behaviors. Moreover, long working hours were found to be related to an excess risk of shift from a normal weight to being overweight [21]. A meta-analysis of Tenk et al. [22] showed a significant association between perceived stress and visceral adiposity as well as lipid parameters of the metabolic syndrome. However, in female nurses, no association was found between burnout and metabolic syndrome, but night shifts, emotional exhaustion and personal accomplishment were related to an increased waist circumference [23]. Long-term spousal caregiving was associated with double the risk of CVD onset [24]. However, the psychological and physiological health impact of caregiving as chronic stress exposure is highly variable, driven largely by the intensity of care provided and the suffering of the care recipient [25,26].

The ‘selfish brain’ theory of Peters et al. [27] was an advance in understanding the central role of the neocortex and limbic system in the pathogenesis of metabolic abnormalities by the expansion of the allostatic load model upon the energetic demands of the brain [28]. Under stressful conditions, the brain behaves in a ‘selfish’ manner and demands extra energy. It actively regulates its own energy supply from the body by using a brain-pull mechanism to satisfy its increased energetic needs prior to other organs. This leads to a ‘cerebral insulin suppression’ to limit glucose fluxes into peripheral tissue, and to enhance cerebral glucose supply. Furthermore, the brain changes eating behaviors in the post-stress phase according to its needs [29,30,31]. People who develop divergent patterns of fat distribution during psychological stress over the course of their life were found. People with high psychological distress combined with low autonomic variability who habituate will be able to tolerate aversive circumstances by buffering the allostatic load, were prone to developing subcutaneous fat accumulation (the corpulent phenotype). However, they did not accumulate much visceral fat, and their cardiovascular mortality risk remains low [28,32]. People who experience the unbuffered allostatic overload of chronic stress belong to the non-habituators that display compromised recovery during a persistent cerebral energy crisis. These neuroenergetic alterations promote visceral fat accumulation (the wide-waisted phenotype), subcutaneous fat loss, and atherogenesis with subsequent cardiovascular events [28,31]. The non-habituators viewed themselves as having less self-esteem, and being more often in a depressed mood than the habituators [33]. Their life-long risk for mental and physical disorders is increased [34]. Therefore, chronic stress without habituation seems to be a key player in the vicious cycle to cardiometabolic disturbances such as visceral adiposity, hyperglycemia, dyslipidemia, and hypertension, collectively named as ‘metabolic syndrome’ with a high risk for CVD [35,36,37,38,39,40,41]. Non-alcoholic fatty liver disease (NAFLD) is strongly related to these cardiometabolic risk factors, but there is also a direct link to chronic psychological stress via increased levels of pro-inflammatory cytokines, cortisol and epinephrine [42,43].

A characteristic body fat distribution plays a key role in the concept of metabolically healthy and unhealthy obese (MHO vs. MUHO) phenotypes. Stefan et al. [44] and Wildman et al. [45] recognized subgroups of individuals with obesity and overweight as having a significantly lower risk of cardiometabolic abnormalities, while a subgroup among normal-weight individuals was surprisingly found to be metabolically unhealthy. Lower amounts of ectopic fat, particularly in the liver and visceral, were observed in the metabolically healthy phenotypes. Moreover, an impaired ability to expand subcutaneous fat resulted in metabolically unhealthy phenotypes [46,47].

Individuals have multidimensional ways to cope with stress. There are aspects of problem-focused and emotion-focused coping strategies [48,49]. Problem-focused stress management strategies attempt to alter the relationship with the environment, whereas emotion-focused stress management strategies aim at reducing, tolerating, or eliminating stress sensations. Negative emotion-focused coping strategies involve the avoidance of stress-related feelings, and preferences for energy-dense foods and overeating, smoking, shorter sleep and a sedentary lifestyle are associated with worse health outcomes later [50]. However, physical activity has the effect of selecting efficient and appropriate coping strategies and reducing the intensity of the stress response [51]. A growing number of studies examined the relationship between psychological stress and cardiometabolic or mental health risk [20]. In the current understanding, there is a missing link, whether high and very high perceived chronic stress loads have a different impact on cardiometabolic dysfunctions highlighting novel therapeutic targets to combat CVD at a population level. Thus, the aim of this study was to investigate the cardiometabolic risk profile in participants with ‘high’ and ‘very high’ chronic stress loads and the impact of positive and negative coping factors used.

## 2. Materials and Methods

### 2.1. Questionnaires: The Perceived Stress Questionnaire (PSQ_30_) and the Psychological Neurological Questionnaire (PNF)

Perceived chronic psychological stress was defined by an elevated level of the German version of the standardized PSQ_30_ [52]. The PSQ_30_ assesses the extent of a subjectively perceived stress in the context of a transactional view of stress [53]. This valid instrument includes 30 items that are applicable to adults of any stage of life, sex, or occupation, but interpretable as specific to a variety of real-life situations. The items are assigned to the seven scales harassment, overload, irritability, lack of joy, fatigue, worries, and tension. The items are worded in such a way that the neutral, cognitive aspect of experience is emphasized and were answered with a four-point rating scale (1 = almost never, 2 = sometimes, 3 = often, and 4 = usually). Mean values for the seven scales were calculated. The total PSQ_30_ score was derived from the raw item scores that were linearly transformed to values between zero (lowest possible level of stress) and one (highest possible level of stress) [53].

The subjective psychological–neurological symptoms of the participants were assessed by the PNF, a questionnaire originally used by occupational medicine [52,54]. It comprises a total of 38 items about the frequency of psychological–neurological symptoms within the past three months. These items are divided into five categories of neurovegetative disturbances: psychoneurovegetative stability, neurological symptoms, impulsion, excitability, concentration, and memory [52,54]. The self-reported symptoms were assessed as total points, including for individual categories ranging from ‘not at all’ (0 points) to ‘often’ (3 points).

### 2.2. Participants

Women and men aged 18 to 65 years with subjectively perceived chronic stress and exhaustion conditions, such as symptoms of burnout, were recruited via local media and online advertising, internal mail systems, flyers and printed media in the Cologne/Bonn region, Germany. At the beginning of the study, all participants had to indicate being progressively exhausted based on the chronic stress experience. Participants with a total PSQ_30_ score above 0.50 were included in the study. Similar to Kocalevent et al. [53], the median of the total PSQ_30_ scores determines the division of the ‘high stress group (HS group)’ and the ‘very high stress group (VHS group)’ in the ITT population.

The exclusion criteria for participation were therapy with antidepressants, hypertension (untreated > 150/90 mmHg, treated > 140/85 mmHg), supplementation or therapy with dietary supplements or drugs that contained L-tryptophan, vitamins, or minerals within four weeks prior to and for the duration of the study, organic fatigue, phenylketonuria, and chronic diarrhea. Informed consent was obtained from each participant prior to the start of the study. The study was approved by the Freiburg International Ethics Commission. This study was retrospectively registered (NCT02997137).

### 2.3. Study Design

This post hoc analysis was performed with the baseline data of the BOT-01 study, a randomized, double-blind, placebo-controlled intervention trial on the impact of a specific amino acid composition with micronutrients on well-being in subjects with chronic psychological stress and exhaustion conditions [52]. The enrolment and baseline examinations were performed at the University Hospital Bonn between October 2014 and January 2016. At baseline, participants underwent an assessment, including detailed anamnesis with questions on individual factors, social environmental factors and problem-focused and/or emotion-focused coping factors. Moreover, the PSQ_30_, the PNF, anthropometric and clinical measurements, blood parameters, salivary samples and dietary intake were assessed. Seven days before baseline, all participants received detailed instructions in written and oral form for taking saliva cortisol samples and accurate recording of dietary intakes. At baseline, after a seven-day pre-randomization run-in period, participants were assigned to one of two intervention groups via computer-generated randomization with a block size of four [52].

### 2.4. Anthropometric and Clinical Measurements

Anthropometric and clinical measurements were performed in the morning after an overnight fasting period of at least 12 h and under standardized conditions. Body weight and height were determined on a personal floor scale with a height measuring stick (MPE-HM, KERN, Balingen, Germany). Body mass index (BMI, kg/m^2^) was calculated. Waist circumference (WC) was measured via flexible tape [52]. After a 10 min resting period in a seated position, three successive measurements of blood pressure (BP) and heart rate were obtained using a digital automatic upper arm BP monitor (model M10-IT, Omron Healthcare Europe B.V., Hoofddorp, The Netherlands) [52]. These accumulated clinical measurements were averaged to determine systolic and diastolic BP and resting heart rate.

### 2.5. Biochemical Analysis

The Laboratory Schottdorf MVZ GmbH (Augsburg, Germany) conducted the laboratory analyses (for further details, see [52]). Saliva samples were collected in the evening (between 10 and 11 p.m.) and in the morning (30 min after awakening) at baseline. The collections were performed according to the laboratory instructions. Participants were instructed not to eat, drink, or smoke for at least 30 min prior to evening saliva collection. Saliva collection in the morning followed an overnight fasting period of at least 12 h. Participants were not allowed to have brushed their teeth before both saliva samples were taken. Therefore, sterile cotton swabs, Cortisol–Salivette^®^ (Sarstedt, Nümbrecht, Germany), were chewed during 30 to 60 s, deposited in collection tubes and subsequently stored in a refrigerator. Salivary cortisol concentrations were determined using Cortisol ELISA (IBL, Hamburg, Germany) and the microplate reader SUNRISE (Tecan, Crailsheim, Germany).

At baseline, venous blood samples were obtained after an overnight fasting period of at least 12 h. The participants received detailed instructions in oral and written form to avoid serotonin- and/or tryptophan-rich foods 2–3 days before blood sampling due to the determination of serum serotonin. Serum glucose (hexokinase method), glycated hemoglobin (HbA1c; turbidimetric immunologic inhibition assay), insulin (ECLIA), gamma-glutamyltransferase (GGT; IFCC method), total cholesterol (TC; CHOD-PAP method), low-density lipoprotein-cholesterol (LDL-C) and high-density lipoprotein-cholesterol (HDL-C; enzymatic color test), triglycerides (TG; GPO-PAP method), and sensitive C-reactive protein (CRP; turbidimetry) were measured using a Roche analyzer. The diagnosis of metabolic syndrome, including individual components such as blood pressure, was made according to the NCEP criteria [41]. Insulin and glucose values were used to calculate the homeostasis model assessment (HOMA) index, which is a measure of insulin resistance. Serum amino acids and serotonin were determined.

The self-reported dietary intake data (three-day food records) were reviewed with each participant. PRODI 6.4 basis software (WVG, Stuttgart, Germany) with database BLS 3.02 was used to calculate the nutrient content of foods. The average of three days was assessed.

### 2.6. Statistical Analysis

Statistical analysis was performed by using SPSS^®^ for Windows (version 24.0, IBM, Armonk, NY, USA). An explorative post hoc analysis was conducted. Data are presented as mean ± standard deviation (SD). Statistical comparisons were performed using the nonparametric Mann–Whitney U test. The exact tests were used due to the small sample size. Differences in classified variables were tested via Fisher’s exact test. The relationship between different parameters and their effects on the metabolism were modelled via parametric linear regression analysis, including age as a covariate. All statistical tests were two-sided. A *p* value of < 0.05 was considered statistically significant.

## 3. Results

### 3.1. Participants

For this analysis, baseline data were available for 62 chronic psychologically stressed participants, of whom 61 participants (43 women and 18 men) were included in the intention-to-treat (ITT) population. One participant had to be excluded from the study due to abnormal blood values such as liver transaminases and CRP. Table 1 shows the characteristics of the participants.

The median of 0.656 of the total PSQ_30_ results of participants determined the assignment to the HS group (total PSQ_30_ score of 0.500–0.656) and the VHS group (total PSQ_30_ score > 0.656). Thus, this group classification caused the significant group difference in the total PSQ_30_ score. The mean total PSQ_30_ score of the VHS group was 1.35 times higher compared to the HS group. In the HS group, there were four times more smokers and significantly less perceived neurovegetative symptoms than in the VHS group. There were no significant group differences in marital status and the number of children. The number of participants who cared for a family member in need or work in shifts was similar in both groups. In the HS group, 32.3% of the participants did no regular physical activity compared to only 13.3% in the VHS group, but without significant group differences.

### 3.2. Anthropometric, Clinical, and Biochemical Parameters

Among all participants, 18.0% were obese, 45.9% had visceral adiposity, 18.0% had elevated blood pressure, and a fatty liver index (FLI) of ≥60 was found in 23.0% (Table 2). In the HS group, visceral adiposity, high blood pressure and metabolic syndrome were more frequent than in the VHS group. Moreover, a significantly higher FLI, LDL-C/HDL-C, and TC/HDL-C quotient were detected in the HS group compared to the VHS group.

**Figure 1:**
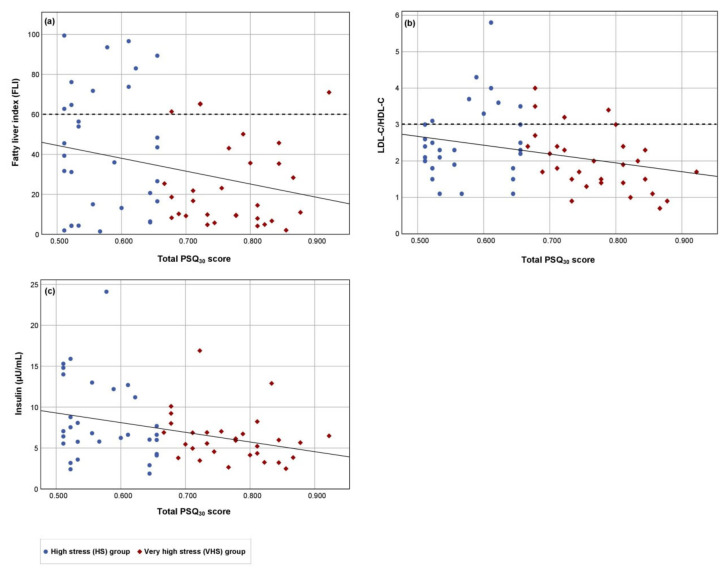
The relationship of the chronic perceived stress load and the cardiometabolic risk in the ITT population. The relationships between the total PSQ_30_ score (independent variable) and the dependent variables (**a**) fatty liver index, (**b**) LDL-C/HDL-C quotient and (**c**) insulin concentration were examined. Results for the high stress (HS) group (*n* = 31), the very high stress (VHS) group (*n* = 30), and the cut-off value (dashed line) for FLI and LDL-C/HDL-C quotient are shown. Regression lines: (**a**) Beta value = −0.266, *p* = 0.038, 95% confidence interval: −125.1 to −3.7; (**b**) Beta value = −0.294, *p* = 0.022, 95% confidence interval: −4.5 to −0.4; (**c**) Beta value = −0.330, *p* = 0.009, 95% confidence interval: −20.6 to −3.0.

In both groups, there was an elevated mean salivary cortisol level in the morning (Table 2). No significant group differences in salivary cortisol, serotonin concentrations (Table 2), serum amino acid concentrations (Table S1), or in dietary energy and nutrients intake (Table S2) were observed. In the ITT population, significant negative correlations were found between the total PSQ_30_ score and the FLI, LDL-C/HDL-C quotient and insulin concentration (Figure 1). These correlations were characterized by high but not very high perceived stress values in combination with data above the laboratory cut-off values. Thus, participants with a high chronic stress load were at higher cardiometabolic risk than participants with a very high chronic stress load (Figure 1). After age adjusting, similar results were observed via linear regression between the total PSQ_30_ score and the FLI (RCB: −63.951, *p* = 0.032; *p* value for age = 0.017), LDL-C/HDL-C quotient (RCB: −2.413, *p* = 0.019; *p* value for age = 0.040) and insulin concentration (RCB: −11.824, *p* = 0.010; *p* value for age = 0.782).

### 3.3. Inflammation, Indirect Markers of Oxidative Stress, Antioxidative Parameters and the Cardiometabolic Risk Profile

No significant difference between the HS group and the VHS group was found in the indirect markers of oxidative stress and inflammation, i.e., ferritin, GGT, CRP, uric acid, folic acid and HbA1c (Table 2). An elevated CRP concentration was observed in both groups (Table 2). Among all participants, CRP, uric acid concentrations, FLI (Table 3), and GGT (Table S3) correlated significantly positive with weight, WC, insulin, and TG concentrations, as well as significantly negative with the HDL-C concentration, while the HbA1c concentration correlated significantly with the FLI (Table 3), but not with CRP (Table 3), GGT (Table S3), and uric acid concentrations (Table 3).

Within both groups, the FLI correlated significantly positive with WC, and TG, as well as significantly negative with the HDL-C concentration (Table 3). However, similar significant correlations of the CRP concentration with WC, TG and HDL-C concentrations were observed only in the HS group (Table 3). Additionally, within the HS group, the FLI correlated significantly with insulin, FPG, and HbA1c concentrations, as well as with the HOMA index, while significant correlations were detected between the FLI and the BP as well as the uric acid concentration only in the VHS group (Table 3). Moreover, CRP concentration correlated significantly with insulin concentration, HOMA index and heart rate only in the VHS group, while uric acid concentration correlated significantly positive with insulin concentration only in the HS group, and positive with HbA1c and negative with HDL-C concentration only in the VHS group (Table 3).

In the ITT population, no significant effect was detected between the nutritional antioxidant intake and the indirect oxidative stress and inflammation markers, except for a positive correlation between the vitamin B_12_ intake and the ferritin concentration (Table S3). However, in the VHS group, a significant correlation between folic acid concentration and FLI was detected (Table 3). No significant association between the total PSQ_30_ score and markers of oxidative stress and inflammation were found (Table S3), except for a negative correlation between the total PSQ_30_ score and the FLI in the ITT population (*p* = 0.032) (Table 3).

### 3.4. Coping Strategies and Cardiometabolic Risk

The comparison between the chronic perceived stress load and the cardiometabolic risk depending on the coping strategies ‘allostatic load’ resulting in visceral adiposity, ‘smoking’, and ‘regular physical activity’ is shown in the ITT population and for both stress groups (Table 4). In the ITT population, participants with visceral adiposity had a higher FLI (*p* < 0.001), a higher HOMA index (*p* = 0.002), and a higher CRP concentration (*p* = 0.024) but a perceived lower chronic stress (*p* = 0.003) than participants without increased visceral fat accumulation (Table 4).

The FLI was higher in participants with visceral adiposity in the HS group (*p* < 0.001) and in the VHS group (*p* = 0.005). No significant group difference in perceived chronic stress was found between participants with and without visceral adiposity within the HS group (*p* = 0.992) and the VHS group (*p* = 0.072) (Table 4). Moreover, participants with visceral adiposity had a significantly higher CRP concentration (*p* = 0.012) and a higher HOMA index (*p* < 0.001) only in the HS group, while a higher HbA1c (*p* = 0.034) was found only in the VHS group (Table 4).

Among all participants, smokers perceived less frequent chronic stress than non-smokers (*p* = 0.008), without a significant group difference in the CRP concentration (*p* = 0.091, Table 4). However, in the VHS group, smokers had a lower HbA1c (*p* = 0.024) and a lower uric acid concentration (*p* = 0.031) than non-smokers (Table 4).

Participants with regular physical activity had a higher HDL-C concentration (*p* = 0.006) and a lower resting heart rate (*p* = 0.008) but perceived more frequent chronic stress (*p* = 0.039) than inactive participants in the ITT population (Table 4). Within both groups, active and inactive participants had a similar chronic stress level, while active participants showed a more favorable HDL-C concentration and resting heart rate only in the HS group (Table 4).

## 4. Discussion

The present study shows that the stress load can be coped with in different ways and that the coping strategy is crucial for cardiometabolic risk. The data indicate that perceiving high chronic stress is significantly associated with the criteria of the metabolic syndrome according to NCEP in the HS group. In contrast, subjects in the VHS group with very high chronic stress perceived psychological–neurological symptoms and neurovegetative disturbances significantly more frequently. As a consequence, at the time of data evaluation, individuals with very high chronic stress had a lower risk of cardiometabolic disturbance. It is postulated that participants with very high chronic stress are more prone to mental illnesses compared to participants with a high chronic stress load. Differences in stress habituation and coping strategies used seemed to be of great importance for the various health risks in these study groups. It is emphasized that very high chronic stress did not decrease the cardiometabolic risk.

Chronic stressors act in a complex way on a person’s mood, behavior, physiological and mental health [6,55]. In the HS group, there were significantly more subjects with visceral adiposity, high liver fat, metabolic syndrome, and elevated blood pressure compared to the VHS group. Thus, different perceived chronic stress loads seemed to have a different impact on visceral fat accumulation and blood pressure, leading to a clearly increased cardiometabolic risk in subjects with high perceived chronic stress. Previous studies showed that perceived stress might have an influence on blood pressure, while no association was found regarding distress [56]. An association between less perceived stress and higher blood pressure levels was noted in several studies [57,58]. Björntorp [59], who provided a basis for understanding how stress mediators damage the cardiovascular system, how habituation protects the vasculature, and why the different fat distribution patterns display diverging prognostic values was used by Peters et al. [28,29,60] in the ‘selfish brain’ theory. In the ‘non-habituators’, psychosocial stress leads to a release of neuropeptide Y (NPY) from sympathetic nerves. NPY and its receptors get up regulated in a glucocorticoid-dependent manner in the visceral fat. This NPY response results in an increase in visceral fat mass in order to supply the brain with extra fuel from an extra-cerebrally located energy depot [28,31,61]. Therefore, chronic stress-induced peripheral NPY plays a mechanistic role in the enhanced vulnerability to visceral obesity, insulin resistance and oxidative stress [36,62]. In the present study, the HS group seemed to be comparable to these ‘non-habituators’ and their phenotypic change as a long-term adaptation to chronic stress via visceral fat accumulation. The ‘habituators’ are characterized as tolerating aversive circumstances by buffering the allostatic load without developing large visceral fat depots. However, they can predominantly accumulate subcutaneous fat under chronic stress, which is their trade-off [28,35,63]. There were fewer participants with visceral adiposity in the present VHS group; conversely, ‘habituators’ were described as experiencing tolerable stress, while ‘non-habituators’ experience toxic stress. However, participants of the VHS group perceived higher chronic stress and a lower cardiometabolic risk than the HS group but suffered neurovegetative disturbances more often. As a consequence, the VHS group cannot be classified as ‘habituators’. Mixed or new phenotypes with cardiometabolic and mental health risk within high chronic stress loads are presumed. In the present HS group, this adaptation to chronic stress seemed to be a negative passive coping strategy to habituate to the high stress load as an initial evolutionary advance in resource management but was hazardous to the cardiometabolic health consequences. In the VHS group, the participants seemed to be more or less protected against such cardiometabolic complications for the moment, but a non-habituation to the toxic stress load with a risk for mental health is presumed. Thus, the present results of the different stress groups can be divided into metabolically healthy and metabolically unhealthy high chronic stress phenotypes following the concept of Stefan et al. [44,64]. These participants seem to have a different susceptibility to visceral adiposity to adapt to the chronic stress load. Due to the fact that both groups had a similar BMI, in the HS group, a loss of subcutaneous fat via SMS-activation and release of free fatty acids into the venous system as well as an increased visceral fat mass by the alteration of the HPA-axis resulting in a shift in body composition is supposed [65,66]. The VHS group seemed to be protected against increased visceral fat accumulation by non-adaptation to the chronic stress load. However, the chronic stress response may lead to a neurobiological ‘slippery slope’ from chronic stress to mental illness, and further, to cardiometabolic diseases [67]. Thus, long-term studies of metabolically healthy vs. metabolically unhealthy individuals with a chronic stress load would provide a timely opportunity for the prevention or earlier specific intervention of stress-related cardiometabolic or mental health risk.

Chronic stress can lead to chronically elevated glucocorticoids that increase the salience of pleasurable or compulsive activities, such as eating high energy ‘comfort food’ via its interaction with the central reward pathways. This circuitry can suppress the HPA axis activation and the feeling of stress [68,69]. A chronically high concentration of glucocorticoids acts systemically with peripheral Neuropeptide Y (NPY), which has anxiolytic activities and acts as the most powerful hunger stimulator in the body to increase abdominal fat depots. These abdominal energy stores send an increased signal to inhibit catecholamines in the brainstem and hypothalamic neurons [68]. Dichotomous effects of chronic stress on metabolic outcomes were found, partly due to the inherent complexity exemplified by the bidirectional effect of stress on eating behaviors and body weight [66]. In total, 25.8% of the participants in the present HS group and 10.0% of the participants in the VHS group are obese, but without group differences. Visceral adiposity was significantly more frequent in the HS group vs. the VHS group. Thus, the prevalence of obesity in the HS group is higher, than in the general German population, with 18.9% of obese men and 22.5% of obese women [70]. No significant difference in the BMI, energy intake, as well as intake of macro- and micronutrients was found between the HS and VHS groups. Thus, the supposed effects of different stress loads on the nutrient intake and emotional eating as a coping strategy were not detected. However, in healthy men with lower chronic stress exposure, a stronger acylated ghrelin response after acute stress exposure compared with a control condition was found, while healthy men with higher chronic stress exposure showed a blunted acylated ghrelin response after acute stress exposure. Consequently, a subsequent food intake is affected differently [71]. Richardson et al. [72] found a positive association between perceived stress and emotional eating but higher stress was not associated with weight status through eating behaviors and diet quality. Geschwind et al. [73] showed that the experience of high daily life rewards can preserve mental health.

In Germany, depressive symptoms, burnout syndrome, and sleep disturbances are more common in people with high levels of chronic stress than without high levels of stress [17]. In the present study, both chronic stress groups suffered from increased psychological neurological symptoms. Participants in the VHS group perceived these neurovegetative disturbances to a higher degree than the HS group. The negative coping strategy smoking was more often evaluated in participants in the HS group than in the VHS group to reduce perceived stress. Smokers were related to lower chronic stress perception than non-smokers. This result confirmed the regulatory role of nicotine in the regulation of stress mediated by the hypothalamus and the reward pathways [74,75] with an increased cardiometabolic health risk [76]. Smoking, the greatest exogenous oxidative stressor [77], is reflected in the increased cardiometabolic risk of the HS group. Moreover, both stress groups used regular physical activity as a positive coping strategy [78,79] to a similar extent. However, participants with regular physical activity perceived a higher stress load than participants without regular physical activity. In general, the experience of stress impairs efforts to be physically active, especially in chronically stressed populations. Nonetheless, stress can positively be impacted by physical activity via behavioral activation through individuals utilizing exercise to cope with stress [80,81].

Thus, participants in the present study, who used physical activity as a coping strategy, had a higher total PSQ_30_ score compared to physically inactive participants with a lower total PSQ_30_ score, who had a higher risk for more frequent negative coping strategies, such as smoking or visceral fat accumulation. The extent of the physical activity level and stress reduction since these participants started with regular physical activity are unknown. An increased risk in gradually replacing regular physical activity by a less time-consuming, negative coping strategy is supposed in individuals with chronic stress. Physical activity is associated with better cardiometabolic outcomes [79,82]. In the present study, regular physical activity was associated with lower resting heart rate and higher HDL-C concentration. Exercise, especially endurance training and yoga, can decrease resting heart rate [83]. A higher resting heart rate is independently associated with an increased risk of cardiovascular mortality [84]. Furthermore, physical activity can improve the antioxidative and anti-inflammatory properties of HDL-C concentration [85].

Chronic stress can lead to low grade inflammation, causing fat accumulation in the liver cells. A persistent inflammation of the liver can result in liver damage. Non-alcoholic fatty liver disease (NAFLD) is a stress-sensitive disorder [86]. A cross-sectional study observed a positive association between perceived stress and NAFLD in apparently healthy women and men [87]. These results indicate that elevated cardiometabolic risk is clearly assigned to participants with a high compared to a very high chronic stress load due to stress coping by visceral fat accumulation. Madhu et al. [88] demonstrated that high chronic stress is associated with a high risk of diabetes mellitus. Subjects with very high chronic stress without visceral adiposity as a stress coping strategy seemed to be protected from this cardiometabolic risk. A relative balance of adaptive and maladaptive coping strategies used is associated with better coping outcomes in the context of chronic illness [89]. However, the healthy brain has a considerable capacity for resilience based upon its ability to respond. Treatments should be given or used by positive behavioral interventions; negative experiences may even make matters worse [90].

In the present study, markers of inflammation and oxidative stress, such as CRP sensitive, GGT, and uric acid were associated with numerous cardiometabolic risk parameters. Both stress groups had increased CRP concentrations. Chronic stressors, such as family dementia caregiving or job strain, promote a state of chronic low-grade elevation in circulating inflammatory markers [91]. Elevations in systemic CRP levels are related to greater incidence of depression, cardiovascular disorders and diabetes, providing a physiological pathway for chronic stress to possibly harm health [92,93,94]. Chronic psychological stress should be considered when interpreting the meaning of CRP elevations [95]. The role of uric acid in cardiometabolic disease is controversial. Hyperuricemia is associated with an increased risk of metabolic syndrome, hypertension, coronary heart disease, insulin resistance, and NAFLD [96,97,98]. Conversely, uric acid plays a protective role in oxidative stress by acting as an active oxygen scavenger with an antioxidant effect, and can prevent cardiovascular diseases (CVD) [97,99]. In the VHS group, uric acid was positively associated with systolic and diastolic blood pressure as well as folic acid concentration. The folic acid concentration was related to FLI in the VHS group. These relationships were not detected in the HS group. Folic acid has a significant inhibition property in microsomal lipid peroxidation, a free radical scavenging behavior [100]. One suggested explanation for the reduced cardiometabolic risk of the VHS group compared to the HS group is a still functioning protective antioxidant capacity, also due to the lower number of smokers in the VHS group. Over time, this condition will possibly change to an adaptation to the HPA-axis dysregulation with increased cardiometabolic risk.

In the VHS group, eight out of thirty subjects had visceral obesity. In the eight participants, visceral adiposity was associated with higher HbA1c and FLI. Moreover, common risk factors of NAFLD [101,102], such as BMI, WC, BP, TG and HDL-C, were related to FLI in the VHS group. A gradual shift of these participants from a very high chronic stress load to a high chronic stress load with higher cardiometabolic risk is presumed. In the HS group, the 20 participants with visceral adiposity had a significant higher FLI, HOMA index, and CRP compared to participants without visceral adiposity. These are already well-known cardiometabolic risk factors associated with visceral adiposity [101]. Moreover, there were significant inverse correlations between the perceived chronic stress and the fatty liver index, LDL-C/HDL-C quotient, and insulin concentration among all participants. As expected, the FLI was associated with WC and insulin, TG, and HDL-C concentrations. All these parameters are related to the metabolic syndrome and increased cardiovascular mortality risk [103]. The meta-analysis of Kuo et al. [104] showed that adults with high stress have an increased risk of having metabolic syndrome than adults with low stress.

The small number of cases and the short study period are limiting factors. Further studies with larger cohorts should investigate different high chronic stress groups, their habituation to stress, and the consequences of cardiometabolic and mental health risks in a long-term study. Further limitations are the lack of a control group, the wide age range of the participants (18 to 65 years of age) and the impossibility of an analysis of sex differences due to the small number of participants.

## 5. Conclusions

These findings indicate that high perceived chronic stress is related to visceral obesity, elevated blood pressure, metabolic syndrome and high liver fat, with a high cardiometabolic risk due to stress-induced disturbances of the HPA axis and maladaptive coping strategies used. On the contrary, a very high perceived chronic stress load seemed to be rather associated with mental health risk than with cardiometabolic risk. Participants in the HS group habituated to their stress load by using coping strategies, such as smoking, and an ‘allostatic load’ that resulted in visceral adiposity in dealing with the perceived chronic stress but was hazardous to the cardiometabolic health consequences. Participants in the VHS group are not currently capable of habituating to their perceived chronic stress but seemed to be cardiometabolically healthier. As expected, inflammation and oxidative stress markers significantly correlated with cardiometabolic risk parameters. Participants using the coping strategy ‘regular physical activity’ had a significantly higher HDL-C concentration and a lower resting heart rate than inactive participants. However, long-term studies are necessary to examine further adaptations to chronic stress in both groups and to evaluate individual stress-management strategies.

## Figures and Tables

**Table 1:** Characteristics of participants.

	ITT Population *n* = 61 Mean ± SD *n* (%)	HS Group ^(1)^ *n* = 31 Mean ± SD *n* (%)	VHS Group ^(2)^ *n* = 30 Mean ± SD *n* (%)	HS vs. VHS *p* Value
Age (years)	44.6 ± 12.1	45.3 ± 12.1	43.7 ± 12.3	0.559
Women	43 (70.5%)	21 (67.7%)	22 (73.3%)	0.780
Men	18 (29.5%)	10 (32.3%)	8 (26.7%)	
Smokers	15 (24.6%)	12 (38.7%)	3 (10.0%)	0.016
Total PSQ_30_ score	0.671 ± 0.118	0.573 ± 0.057	0.771 ± 0.069	<0.001
PNF (total)	51.3 ± 12.1	46.2 ± 10.9	56.6 ± 11.2	<0.001
**Marital status ^(3)^:**				
Married/cohabitation	31 (50.8%)	14 (45.2%)	17 (56.7%)	0.373
Single	17 (27.9%)	11 (35.5%)	6 (20.0%)	
Separated	12 (19.7%)	6 (19.4%)	6 (20.0%)	
**Children ^(3)^:**				
0 children	32 (52.5%)	16 (51.6%)	16 (53.3%)	0.445
1 child	15 (24.6%)	10 (32.3%)	5 (16.7%)	
2 children	9 (14.8%)	3 (9.7%)	6 (20.0%)	
≥3 children	4 (6.6%)	2 (6.5%)	2 (6.7%)	
**Care for a family member in need ^(4)^:**				
Yes	17 (27.9%)	9 (29.0%)	8 (26.7%)	1.000
No	42 (68.9%)	21 (67.7%)	21 (70.0%)	
**Work in shifts:**				
Yes	14 (23.0%)	6 (19.4%)	8 (26.7%)	0.554
No	47 (77.0%)	25 (80.6%)	22 (73.3%)	
**Regular physical activity:**				
No	14 (23.0%)	10 (32.3%)	4 (13.3%)	0.127
Yes	47 (77.0%)	21 (67.7%)	26 (86.7%)	
Yes: athletic and endurance sports (jogging, bicycling, swimming, fitness studio or horse-riding)	20 (32.8%)	8 (25.8%)	12 (41.4%)	0.283
Yes: low-intensity endurance sports (Nordic walking or going for a walk)	21 (34.4%)	9 (29.0%)	12 (40.0%)	0.426
Yes: autogenous training or yoga	6 (9.8%)	4 (12.9%)	2 (6.7%)	0.671

Abbreviations: IIT, intention-to-treat population; PNF, Psychological Neurological Questionnaire; PSQ, Perceived Stress Questionnaire; SD, standard deviation. ^(1)^ High stress (HS) group: participants with a total PSQ_30_ score of 0.500–0.656; ^(2)^ Very high stress (VHS) group: participants with a total PSQ_30_ score > 0.656; ^(3)^ one person did not answer the question because of personal reasons; ^(4)^ two persons did not answer the question because of personal reasons; *p* Value: Mann–Whitney U test or Fisher’s exact test.

**Table 2:** Cardiometabolic risk of participants.

	ITT Population *n* = 61 Mean ± SD *n* (%)	HS Group ^(1)^ *n* = 31 Mean ± SD *n* (%)	VHS Group ^(2)^ *n* = 30 Mean ± SD *n* (%)	HS vs. VHS *p* Value
**Anthropometric and clinical parameters**				
BMI (kg/m^2^)	25.5 ± 5.1	26.6 ± 6.0	24.4 ± 3.71	0.169
WC (cm)_women	86.3 ± 12.4	89.7 ± 14.3	83.4 ± 9.44	0.079
WC (cm)_men	101.7 ± 13.6	106.1 ± 16.3	96.2 ± 6.67	0.051
BP systolic (mmHg)	112.1 ± 14.2	115.2 ± 14.7	109.0 ± 13.1	0.094
BP diastolic (mmHg)	75.2 ± 8.5	76.6 ± 8.7	73.7 ± 8.2	0.141
Resting heart rate (1/min)	66.5 ± 8.5	66.2 ± 7.7	66.9 ± 9.5	0.906
**Cardiometabolic risk parameters**				
BMI ≥ 30.0 kg/m^2^	11 (18.0%)	8 (25.8%)	3 (10.0%)	0.182
WC ≥ 88 cm (w/≥ 102 cm (m))	28 (45.9%)	20 (64.5%)	8 (26.7%)	0.005
BP ≥ 130/85 mmHg	11 (18.0%)	9 (29.0%)	2 (6.7%)	0.043
TC (mg/dL)	212.2 ± 46.9	217.0 ± 45.4	207.2 ± 48.8	0.372
TC > 200	35 (57.4%)	21 (67.7%)	14 (46.7%)	0.124
HDL-C (mg/dL)	64.7 ± 19.8	60.2 ± 17.1	69.4 ± 21.6	0.117
HDL-C < 50 (w)/< 40 (m)	14 (23.0%)	7 (22.6%)	7 (23.3%)	1.000
LDL-C (mg/dL)	134.2 ± 41.6	140.1 ± 39.4	128.1 ± 43.6	0.261
LDL-C ≥ 175	11 (18.0%)	6 (19.4%)	5 (16.7%)	1.000
TG (mg/dL)	100.1 ± 53.7	111.8 ± 68.4	88.1 ± 28.9	0.249
TG ≥ 150	6 (9.8%)	5 (16.1%)	1 (3.3%)	0.195
Fasting plasma glucose (mg/dL)	89.7 ± 17.1	91.7 ± 22.2	87.7 ± 9.2	0.923
FPG ≥ 100 (mg/dL)	7 (11.5%)	5 (16.1%)	2 (6.7%)	0.425
HbA1c (%)	5.5 ± 0.6	5.6 ± 0.7	5.4 ± 0.3	0.179
HOMA index	1.7 ± 1.5	2.1 ± 2.0	1.3 ± 0.6	0.184
Insulin–ECLIA (μU/mL)	7.3 ± 4.2	8.3 ± 4.9	6.2 ± 3.0	0.107
Metabolic syndrome (NCEP [41])	6 (9.8%)	6 (19.4%)	0 (0%)	0.024
ALAT (U/L)	23.7 ± 12.0	24.94 ± 12.8	22.5 ± 11.1	0.540
ASAT (U/L)	25.2 ± 12.6	26.8 ± 17.2	23.5 ± 4.1	0.860
FLI	33.5 ± 28.4	42.5 ± 31.9	24.1 ± 21.1	0.045
FLI ≥ 30	28 (45.9%)	19 (61.3%)	9 (30.0%)	0.021
FLI ≥ 60	14 (23.0%)	10 (32.3%)	4 (13.3%)	0.127
Atherogenic dyslipoproteinemia	3 (4.9%)	3 (9.7%)	0 (0%)	0.238
TC/HDL-C	3.5 ± 1.2	3.9 ± 1.4	3.2 ± 0.9	0.022
LDL-C/HDL-C	2.3 ± 1.0	2.5 ± 1.0	2.0 ± 0.8	0.030
**Serum serotonin and salivary cortisol**				
Serum serotonin (μg/L)	144.3 ± 69.2	135.4 ± 47.8	153.4 ± 85.8	0.734
Cortisol_morning (ng/mL)	13.8 ± 5.7	14.0 ± 6.0	13.6 ± 5.5	0.783
Cortisol_evening (ng/mL)	2.5 ± 3.8	2.7 ± 3.7	2.3 ± 3.9	0.544
Δ cortisol (m–e) (ng/mL)	11.3 ± 6.0	11.3 ± 6.9	11.3 ± 5.1	0.997
**Oxidative stress markers and antioxidative status**				
CRP sensitive (mg/L)	2.2 ± 3.4	2.0 ± 2.9	2.4 ± 3.9	0.821
Ferritin (ng/mL)	114.9 ± 106.9	116.6 ± 132.5	113.1 ± 74.2	0.458
GGT (U/L)	22.2 ± 13.2	25.1 ± 15.0	19.3 ± 10.6	0.142
Folic acid (ng/mL) ^(3)^	9.6 ± 3.6	9.4 ± 3.8	9.8 ± 3.4	0.438
Uric acid (mg/dL)	4.8 ± 1.1	5.0 ± 1.1	4.6 ± 1.1	0.179

Abbreviations: ALAT, alanine aminotransferase; ASAT, aspartate aminotransferase; BMI, body mass index; BP, blood pressure; Δ cortisol (m–e), cortisol difference morning–evening; CRP, C-reactive protein; ECLIA, electrochemiluminescence immunoassay; FLI, fatty liver index; FPG, fasting plasma glucose; GGT, gamma-glutamyltransferase; HbA1c, glycated hemoglobin A1c; HDL-C, high-density lipoprotein cholesterol; HOMA index, homeostasis model assessment index; ITT, intention-to-treat population; LDL-C, low-density lipoprotein cholesterol; NCEP, National Cholesterol Education Program; SD, standard deviation; TC, total cholesterol; TG, triglycerides; WC, waist circumference. ^(1)^ High stress (HS) group: participants with a total PSQ_30_ score of 0.500–0.656; ^(2)^ Very high stress (VHS) group: participants with a total PSQ_30_ score > 0.656; ^(3)^ *n* = 60, data not available for one participant (hemolytic blood sample); *p* Value: Mann–Whitney U test or Fisher’s exact test.

**Table 3:** Relationship of cardiometabolic risk parameters and in-/direct markers of inflammation and oxidative stress (age adjusted).

	CRP (mg/L)						Uric Acid (mg/dL)						FLI					
	ITT Population		HS Group ^(1)^		VHS Group ^(2)^		ITT Population		HS Group ^(1)^		VHS Group ^(2)^		ITT Population		HS Group ^(1)^		VHS Group ^(2)^	
	RCB	*p* Value	RCB	*p* Value	RCB	*p* Value	RCB	*p* Value	RCB	*p* Value	RCB	*p* Value	RCB	*p* Value	RCB	*p* Value	RCB	*p* Value
Weight (kg)	0.054	0.033	0.054	0.057	0.073	0.160	0.038	<0.001	0.036	0.001	0.040	0.003	1.366	<0.001	1.298	<0.001	1.255	<0.001
WC (cm)	0.066	0.045	0.081	0.022	0.072	0.328	0.039	<0.001	0.036	0.010	0.045	0.020	1.805	<0.001	1.720	<0.001	1.722	<0.001
BMI (kg/m^2^)	0.204	0.020	0.185	0.052	0.370	0.072	0.090	0.001	0.082	0.029	0.101	0.070	4.694	<0.001	4.228	<0.001	5.053	<0.001
Syst. BD (mmHg)	0.019	0.568	−0.010	0.810	0.055	0.342	0.036	<0.001	0.019	0.262	0.052	<0.001	0.834	0.001	0.513	0.216	0.726	0.014
Diast. BD (mmHg)	0.058	0.277	0.009	0.894	0.121	0.189	0.041	0.017	0.011	0.689	0.065	0.006	1.428	<0.001	1.089	0.086	1.249	0.008
Heart rate (1/min)	0.108	0.038	0.003	0.965	0.180	0.021	0.012	0.497	0.004	0.890	0.016	0.466	0.812	0.049	1.179	0.084	0.426	0.326
Insulin (μU/mL)	0.279	0.007	0.158	0.157	0.779	0.001	0.095	0.005	0.091	0.036	0.082	0.245	3.738	<0.001	3.773	<0.001	1.524	0.271
HOMA index	0.494	0.088	0.283	0.294	3.495	0.002	0.250	0.007	0.202	0.054	0.509	0.118	9.889	<0.001	8.583	<0.001	8.555	0.184
FPG (mg/dL)	0.017	0.527	0.016	0.530	0.038	0.670	0.022	0.013	0.017	0.077	0.043	0.072	0.801	<0.001	0.767	<0.001	0.619	0.190
HbA1c (%)	0.454	0.572	0.373	0.613	2.447	0.451	0.437	0.093	0.274	0.345	1.703	0.046	17.633	0.004	15.368	0.025	27.970	0.100
TG (mg/dL)	0.027	0.001	0.031	<0.001	0.032	0.223	0.007	0.006	0.006	0.076	0.013	0.075	0.290	<0.001	0.226	0.003	0.354	0.008
HDL-C (mg/dL)	−0.066	0.004	−0.086	0.004	−0.068	0.073	−0.029	<0.001	−0.022	0.079	−0.033	0.001	−0.903	<0.001	−1.100	<0.001	−0.548	0.005
Serotonin (μg/L)	−0.006	0.399	−0.015	0.214	−0.003	0.704	−0.005	0.029	−0.008	0.075	−0.003	0.199	−0.112	0.029	−0.082	0.492	−0.088	0.560
Vitamin E (mg)	0.061	0.338	0.029	0.746	0.076	0.436	0.029	0.165	0.024	0.507	0.034	0.198	0.512	0.308	0.374	0.671	0.426	0.417
Vitamin C (mg)	0.002	0.658	−0.005	0.639	0.004	0.575	0.001	0.398	0.002	0.651	0.002	0.385	0.029	0.486	−0.004	0.965	0.049	0.187
Folic acid (ng/mL) ^(3)^	−0.049	0.705	−0.257	0.076	0.202	0.386	0.001	0.349	−0.017	0.768	0.120	0.044	0.980	0.340	0.140	0.923	2.397	0.043
PFA (g)	−0.013	0.835	0.016	0.864	−0.044	0.644	0.025	0.206	0.037	0.304	0.025	0.329	0.498	0.308	0.594	0.499	0.460	0.362
Vitamin B_12_ (µg)	0.034	0.789	0.267	0.100	−0.136	0.511	0.066	0.114	0.156	0.012	−0.022	0.697	1.917	0.055	3.714	0.015	−0.065	0.954
Total PSQ_30_ score	−0.008	0.998	−1.960	0.848	−8.574	0.439	−1.918	0.123	−6.943	0.078	1.049	0.729	−63.95	0.032	−102.2	0.297	16.23	0.785

Abbreviations: BMI, body mass index; BP (syst./diast.), systolic or diastolic blood pressure; CRP, C-reactive protein sensitive; FLI, fatty liver index; FPG, fasting plasma glucose; HbA1c, glycated hemoglobin A1c; HDL-C, high-density lipoprotein cholesterol; HOMA index, homeostasis model assessment index; ^(1)^ High stress (HS) group: participants with a total PSQ_30_ score of 0.500–0.656; ^(2)^ Very high stress (VHS) group: participants with a total PSQ_30_ score > 0.656. ITT, intention-to-treat population; PFA, polyunsaturated fatty acids; RCB, regression coefficient B; TG, triglycerides; WC, waist circumference. ^(3)^ *n* = 60, data not available for one participant (hemolytic blood sample); *p* value: linear regression analysis.

**Table 4:** Coping strategies and cardiometabolic health of participants.

	ITT Population Mean ± SD			HS Group ^(1)^ Mean ± SD			VHS Group ^(2)^ Mean ± SD		
**Coping strategy: ‘Allostatic load’ resulting in visceral adiposit**									
	**With** **visceral** **adiposity** ***n* = 28**	**Without** **visceral** **adiposity** ***n* = 33**	***p* value**	**With** **visceral** **adiposity** ***n* = 20**	**Without** **visceral** **adiposity** ***n* = 11**	***p* value**	**With** **visceral** **adiposity** ***n* = 8**	**Without** **visceral** **adiposity** ***n* = 22**	***p* value**
PSQ_30_ score	0.619 ± 0.093	0.714 ± 0.120	0.003	0.573 ± 0.058	0.574 ± 0.058	0.992	0.735 ± 0.053	0.784 ± 0.070	0.072
FLI	51.8 ± 25.8	17.9 ± 20.3	<0.001	56.1 ± 26.7	17.7 ± 25.5	<0.001	40.9 ± 21.2	18.1 ± 17.9	0.005
HOMA index	2.3 ± 2.1	1.2 ± 0.5	0.002	2.7 ± 2.3	0.9 ± 0.4	<0.001	1.4 ± 0.9	1.3 ± 0.5	0.738
HbA1c (%)	5.7 ± 0.8	5.4 ± 0.3	0.050	5.7 ± 0.9	5.5 ± 0.3	0.830	5.6 ± 0.2	5.3 ± 0.3	0.034
CRP (mg/L)	2.6 ± 3.6	1.8 ± 3.3	0.024	2.1 ± 2.0	1.8 ± 4.2	0.012	3.9 ± 6.0	1.8 ± 2.8	0.510
**Coping strategy: Smoking**									
	**Smokers** ***n* = 15**	**Non-** **smokers** ***n* = 46**	***p* value**	**Smokers** ***n* = 12**	**Non-** **smokers** ***n* = 19**	***p* value**	**Smokers** ***n* = 3**	**Non-** **smokers** ***n* = 27**	***p* value**
PSQ_30_ score	0.602 ± 0.091	0.693 ± 0.118	0.008	0.567 ± 0.054	0.578 ± 0.060	0.437	0.744 ± 0.062	0.774 ± 0.070	0.563
HbA1c (%)	5.7 ± 1.0	5.4 ± 0.3	0.643	5.9 ± 1.1	5.4 ± 0.3	0.270	5.1 ± 0.1	5.4 ± 0.3	0.024
Uric acid (mg/dL)	4.8 ± 1.1	4.8 ± 1.1	0.990	5.2 ± 1.0	4.9 ± 1.2	0.515	3.4 ± 0.3	4.7 ± 1.1	0.031
CRP (mg/L)	3.1 ± 3.8	1.9 ± 3.3	0.091	3.5 ± 4.2	1.1 ± 0.8	0.071	1.6 ± 1.2	2.5 ± 4.1	0.778
**Coping strategy: Physical activity**									
	**With** **physical** **activity** ***n* = 47**	**Without** **physical** **activity** ***n* = 14**	***p* value**	**With** **physical** **activity** ***n* = 21**	**Without** **physical** **activity** ***n* = 10**	***p* value**	**With** **physical** **activity** ***n* = 26**	**Without** **physical** **activity** ***n* = 4**	***p* value**
PSQ_30_ score	0.687 ± 0.116	0.614 ± 0.109	0.039	0.581 ± 0.058	0.558 ± 0.052	0.306	0.773 ± 0.069	0.755 ± 0.076	0.670
HbA1c (%)	5.4 ± 0.3	5.8 ± 1.0	0.060	5.4 ± 0.3	6.0 ± 1.2	0.194	5.4 ± 0.3	5.5 ± 0.2	0.293
HDL-C (mg/dL)	67.5 ± 17.4	55.2 ± 24.8	0.006	64.5 ± 16.9	51.1 ± 14.3	0.030	70.0 ± 17.7	65.4 ± 43.1	0.298
Resting heart rate (1/min)	65.0 ± 8.5	71.6 ± 6.7	0.008	63.6 ± 6.7	71.7 ± 6.8	0.006	66.2 ± 9.7	71.3 ± 7.4	0.305

Abbreviations: CRP, C-reactive protein sensitive; FLI, fatty liver index; HbA1c, glycated hemoglobin A1c; HDL-C, high-density lipoprotein cholesterol; HOMA index, homeostasis model assessment index; ITT, intention-to-treat population; PSQ, Perceived Stress Questionnaire; SD, standard deviation; ^(1)^ High stress group: participants with a total PSQ_30_ score of 0.500–0.656; ^(2)^ Very high stress group: participants with a total PSQ_30_ score > 0.656; *p* value: Mann–Whitney U test.

## Data Availability

The data presented in this study are available on request.

## Supplementary Materials

The following are available online at https://www.mdpi.com/article/10.3390/nu14010077/s1, Table S1: Serum amino acid concentrations of participants, Table S2: Dietary intake of participants, Table S3: Relationship of cardiometabolic risk parameters and GGT as well as ferritin concentrations in the ITT population (age-adjusted).
