# Supplementary material for: Coping Strategies Influence Cardiometabolic Risk Factors in Chronic Psychological Stress: A Post Hoc Analysis of A Randomized Pilot Study
Source: Nutrients. 2021 Dec 24;14(1):77. doi: 10.3390/nu14010077 (PMC8747048; doi:10.3390/nu14010077)
Supplement: Supplementary file 1 [file nutrients-14-00077-s001.zip › Table S1. Serum amino acid concentrations of participants.pdf]

Table S1. Serum amino acid concentrations of participants.

	ITT population<br>n = 61<br>mean ± SD<br>n (%)	HS-group[(1)]<br>n = 31<br>mean ± SD<br>n (%)	VHS-group[(2)]<br>n = 30<br>mean ± SD<br>n (%)	HS vs. VHS<br>*p* – Value
L-ornithine (µmol/L)	102.8 ± 27.5	106.1 ± 29.7	99.4 ± 25.0	0.210
L-phenylalanine (µmol/L)	97.5 ± 16.7	99.8 ± 15.2	95.2 ± 18.1	0.530
Taurine (µmol/L)	155.1 ± 31.1	152.1 ± 25.6	158.3 ± 36.1	0.784
L-tryptophan (µmol/L)	54.1 ± 12.1	54.8 ± 10.7	53.3 ± 13.6	0.414
L-tyrosine (µmol/L)	68.6 ± 15.1	69.1 ± 18.1	68.2 ± 11.6	0.498

Abbreviations: Intention-to-treat (ITT) population; SD, standard deviation; [(1)] High stress group: participants with a total PSQ_{30}-score of 0.500-0.656; [(2)] Very high stress group: participants with a total PSQ_{30}-score > 0.656; p-Value: Mann–Whitney U test.
