# Supplementary material for: Coping Strategies Influence Cardiometabolic Risk Factors in Chronic Psychological Stress: A Post Hoc Analysis of A Randomized Pilot Study
Source: Nutrients. 2021 Dec 24;14(1):77. doi: 10.3390/nu14010077 (PMC8747048; doi:10.3390/nu14010077)
Supplement: Supplementary file 1 [file nutrients-14-00077-s001.zip › Table S2. Dietary intake of participants.pdf]

Table S2. Dietary intake of participants.

	**ITT population n = 61 mean ± SD n (%)**	**HS-group[(1)] n = 31 mean ± SD n (%)**	**VHS-group[(2)] n = 30 mean ± SD n (%)**	**HS vs. VHS *p*–Value**
Energy (kcal)	2322.6 ± 644.3	2335.2 ± 611.2	2309.5 ± 687.1	0.937
Carbohydrates (g)	238.4 ± 67.5	233.4 ± 67.8	243.5 ± 68.0	0.542
Protein (g)	90.5 ± 32.8	91.3 ± 28.1	89.7 ± 37.5	0.581
Fat (g)	100.3 ± 37.0	103.3 ± 35.4	97.2 ± 38.9	0.378
SFA (g)	42.8 ± 17.9	45.2 ± 18.9	40.4 ± 16.8	0.285
MFA (g)	34.9 ± 13.3	36.5 ± 13.0	33.2 ± 13.6	0.243
PFA (g)	15.4 ± 7.3	14.3 ± 6.3	16.6 ± 8.2	0.259
Alcohol (g)	6.3 ± 10.8	7.2 ± 13.7	5.2 ± 6.5	0.969
Dietary fibers (g)	22.2 ± 9.7	19.7 ± 7.2	24.7 ± 11.4	0.092
Magnesium (mg)	381.6 ± 138.1	370.6 ± 91.6	392.9 ± 174.7	0.914
Folic acid (µg)	272.1 ± 126.2	262.3 ± 94.9	282.3 ± 153.0	0.914
Iron (mg)	13.4 ± 4.7	13.3 ± 3.5	13.6 ± 5.8	0.799
Vitamin B_{12} (µg)	6.0 ± 3.6	6.7 ± 3.4	5.4 ± 3.8	0.085
Vitamin E (mg)	14.3 ± 7.1	13.6 ± 6.1	14.9 ± 8.0	0.739
Vitamin C (mg)	122.8 ± 87.3	115.3 ± 58.2	130.6 ± 110.1	0.791
Zinc (mg)	12.3 ± 4.5	12.4 ± 3.9	12.3 ± 5.2	0.581
Tryptophan (mg)	1078.0 ± 395.5	1089.0 ± 346.9	1066.7 ± 445.9	0.683
Cholesterol (mg)	413.1 ± 228.1	433.4 ± 242.8	392.1 ± 213.9	0.726
Biotin (µg)	53.9 ± 32.8	52.5 ± 17.9	55.5 ± 43.4	0.611
ß-carotene (µg)	4670.2 ± 4289.4	4083.4 ± 3585.1	5276.5 ± 4901.3	0.285

Abbreviations: Intention-to-treat (ITT) population; MFA, monounsaturated fatty acids; PFA, polyunsaturated fatty acids; SD, standard deviation; SFA, saturated fatty acids; [(1)] High stress group: participants with a total PSQ_{30}-score of 0.500-0.656; [(2)] Very high stress group: participants with a total PSQ_{30}-score > 0.656; p-Value: Mann–Whitney U test.
