# Supplementary material for: Coping Strategies Influence Cardiometabolic Risk Factors in Chronic Psychological Stress: A Post Hoc Analysis of A Randomized Pilot Study
Source: Nutrients. 2021 Dec 24;14(1):77. doi: 10.3390/nu14010077 (PMC8747048; doi:10.3390/nu14010077)
Supplement: Supplementary file 1 [file nutrients-14-00077-s001.zip › Table S3. Relationship of cardiometabolic risk parameters.pdf]

Table S3. Relationship of cardiometabolic risk parameters and GGT as well as ferritin concentrations in the ITT population (age-adjusted).

	**GGT (U/L)**						**Ferritin (ng/mL)**					
	**ITT population**		**HS-group [(1)]**		**VHS-group [(2)]**		**ITT population**		**HS-group [(1)]**		**VHS-group [(2)]**	
	**RCB**	***p*- Value**	**RCB**	***p*- Value**	**RCB**	***p*- Value**	**RCB**	***p*- Value**	**RCB**	***p*- Value**	**RCB**	***p*- Value**
Weight (kg)	0.291	**0.002**	0.285	**0.037**	0.215	0.117	2.626	**<0.001**	3.014	**0.016**	2.554	**0.004**
WC (cm)	0.338	**0.005**	0.320	0.068	0.223	0.257	2.880	**0.003**	3.527	**0.028**	2.682	**0.037**
BMI (kg/m^2)	0.620	0.060	0.412	0.387	0.569	0.310	5.833	**0.030**	5.540	0.207	9.186	**0.010**
Syst. BD (mmHg)	0.188	0.123	0.009	0.965	0.234	0.124	1.439	0.150	1.595	0.413	1.600	0.119
Diast. BD (mmHg)	0.340	0.086	0.074	0.821	0.460	0.056	0.836	0.611	0.109	0.971	1.606	0.335
Heart rate (1/min)	0.169	0.390	0.062	0.861	0.210	0.331	-0.644	0.689	-2.064	0.526	0.256	0.862
Insulin (µU/mL)	0.948	**0.015**	0.889	0.100	0.477	0.492	3.464	0.287	3.015	0.556	5.921	0.201
HOMA-index	2.764	**0.009**	2.313	0.073	3.096	0.339	10.727	0.226	9.364	0.445	32.821	0.128
FPG (mg/dL)	0.227	**0.022**	0.208	0.081	0.228	0.337	1.164	0.156	1.095	0.331	1.714	0.282
HbA1c (%)	5.386	0.068	4.769	0.177	7.092	0.411	23.760	0.331	18.371	0.581	91.487	0.110
TG (mg/dL)	0.094	**0.002**	0.053	0.187	0.241	**<0.001**	0.343	0.185	0.313	0.409	0.683	0.146
HDL-C (mg/dL)	-0.225	**0.008**	-0.177	0.253	-0.201	**0.047**	-1.838	**0.008**	-2.667	0.058	-1.538	**0.022**
Serotonin (µg/L)	-0.002	0.934	0.061	0.309	-0.009	0.695	-0.248	0.215	0.277	0.619	-0.399	**0.008**
Vitamin E (mg)	0.443	0.058	0.660	0.128	0.307	0.237	2.413	0.211	4.878	0.229	1.039	0.557
Vitamin C (mg)	0.017	0.367	-0.032	0.497	0.035	0.054	0.183	0.247	0.109	0.802	0.206	0.092
Folic acid (ng/mL) [(3)]	0.313	0.518	-0.726	0.313	1.744	**0.002**	5.047	0.200	2.637	0.695	8.107	**0.043**
PFA (g)	0.164	0.474	0.356	0.419	0.081	0.748	2.971	0.111	7.013	0.080	0.919	0.590
Vitamin B_{12} (µg)	0.812	0.084	1.281	0.105	0.275	0.618	11.491	**0.002**	21.644	**0.002**	3.714	0.314
Total PSQ_{30}-score	-20.572	0.146	-36.552	0.460	4.482	0.880	38.359	0.743	532.032	0.243	-101.584	0.611

Abbreviations: BMI, body mass index; BP (syst./diast.), systolic or diastolic blood pressure; CRP, C-reactive protein sensitive; FLI, fatty liver index; FPG, fasting plasma glucose; GGT, gamma-glutamyl-transferase; HbA1c, glycated hemoglobin A1c; HDL-C, high-density lipoprotein cholesterol; HOMA-index, homeostasis model assessment index; [(1)] High stress (HS)-group: participants with a total PSQ_{30}-score of 0.500-0.656; [(2)] Very high stress (VHS)-group: participants with a total PSQ_{30}-score > 0.656; Intention-to-treat (ITT) population; PFA, polyunsaturated fatty acids; PSQ, Perceived Stress Questionnaire; RCB, regression coefficient B; TG, triglycerides; WC, waist circumference; [(3)] n = 60, data not available for one participant (hemolytic blood sample); *p*-Value: linear regression analysis.
